# Metabolic Programming during Lactation Stimulates Renal Na^+^ Transport in the Adult Offspring Due to an Early Impact on Local Angiotensin II Pathways

**DOI:** 10.1371/journal.pone.0021232

**Published:** 2011-07-01

**Authors:** Ricardo Luzardo, Paulo A. Silva, Marcelo Einicker-Lamas, Susana Ortiz-Costa, Maria da Graça Tavares do Carmo, Leucio D. Vieira-Filho, Ana D. O. Paixão, Lucienne S. Lara, Adalberto Vieyra

**Affiliations:** 1 Institute of Biophysics Carlos Chagas Filho, Federal University of Rio de Janeiro, Rio de Janeiro, Brazil; 2 National Institute for Structural Biology and Bioimaging, Federal University of Rio de Janeiro, Rio de Janeiro, Brazil; 3 Institute of Nutrition Josué de Castro, Federal University of Rio de Janeiro, Rio de Janeiro, Brazil; 4 Department of Physiology and Pharmacology, Federal University of Pernambuco, Recife, Brazil; 5 Institute of Biomedical Sciences, Federal University of Rio de Janeiro, Rio de Janeiro, Brazil; Universidade de Sao Paulo, Brazil

## Abstract

**Background:**

Several studies have correlated perinatal malnutrition with diseases in adulthood, giving support to the programming hypothesis. In this study, the effects of maternal undernutrition during lactation on renal Na^+^-transporters and on the local angiotensin II (Ang II) signaling cascade in rats were investigated.

**Methodology/Principal Findings:**

Female rats received a hypoproteic diet (8% protein) throughout lactation. Control and programmed offspring consumed a diet containing 20% protein after weaning. Programming caused a decrease in the number of nephrons (35%), in the area of the Bowman's capsule (30%) and the capillary tuft (30%), and increased collagen deposition in the cortex and medulla (by 175% and 700%, respectively). In programmed rats the expression of (Na^+^+K^+^)ATPase in proximal tubules increased by 40%, but its activity was doubled owing to a threefold increase in affinity for K^+^. Programming doubled the ouabain-insensitive Na^+^-ATPase activity with loss of its physiological response to Ang II, increased the expression of AT_1_ and decreased the expression of AT_2_ receptors), and caused a pronounced inhibition (90%) of protein kinase C activity with decrease in the expression of the α (24%) and ε (13%) isoforms. Activity and expression of cyclic AMP-dependent protein kinase decreased in the same proportion as the AT_2_ receptors (30%). *In vivo* studies at 60 days revealed an increased glomerular filtration rate (GFR) (70%), increased Na^+^ excretion (80%) and intense proteinuria (increase of 400% in protein excretion). Programmed rats, which had normal arterial pressure at 60 days, became hypertensive by 150 days.

**Conclusions/Significance:**

Maternal protein restriction during lactation results in alterations in GFR, renal Na^+^ handling and in components of the Ang II-linked regulatory pathway of renal Na^+^ reabsorption. At the molecular level, they provide a framework for understanding how metabolic programming of renal mechanisms contributes to the onset of hypertension in adulthood.

## Introduction

Undernutrition is a worldwide public health issue affecting more than one billion people, particularly in underdeveloped countries, where 25% of the undernourished population is children [1]. Therefore, several recent studies have sought to correlate undernutrition in critical periods of development (gestation and/or lactation) with various diseases in adulthood. Studies supporting the programming hypothesis [2], [3], [4], [5] have demonstrated that adverse fetal or neonatal environments such as undernutrition result in adaptative responses leading to structural and molecular alterations in various organs and tissues. The persistence of these modifications results in the development of several diseases in adult life, particularly affecting the cardiovascular and renal systems. These pathological situations are frequently associated with hypertension [6].

The precise mechanism(s) involved in increased blood pressure as a late consequence of metabolic programming is as yet unclear. Most experimental data indicate that hypertension is multifactorial and involves alterations in various organs including the kidney [7], [8]. Kidneys play a major role in the long-term control of arterial blood pressure by regulating Na^+^ intake/excretion [9]. It has been reported that offspring from rats that are protein-restricted throughout gestation present with marked oligonephroenia (a decrease in the number of nephrons), which can lead to a reduction in pressure natriuresis and consequent elevation of blood pressure [10], [11]. However, the reduced number of nephrons is not the sole cause of hypertension in the protein restriction model of programming [6], [12]. The intrarenal molecular machinery may also be altered, contributing to the programming of hypertension [12].

Numerous experimental studies support the view that impaired tubular Na^+^ reabsorption constitutes an important renal modification in hypertensive subjects [13] and spontaneously hypertensive rats [14]. Therefore, inappropriate functioning of Na^+^ transporters as the result of metabolic programming could be one of the intrinsic renal defects that contribute to alterations in Na^+^ handling, leading to adult hypertension. Using kidneys from the offspring of female rats that had been malnourished during pregnancy, Bertram and coworkers [15] observed an increase in mRNA expression of (Na^+^+K^+^)ATPase α_1_ and β_1_ subunits. In addition, transcriptional up-regulation and protein expression of two specific Na^+^ transporters, located in the thick ascending limb of Henle's loop and the distal convoluted tubule, were evident in offspring from female rats exposed to a low-protein diet during gestation [16]. These alterations could result in increased fluid reabsorption and expansion of the intravascular compartment.

Increased tubular Na^+^ reabsorption resulting from altered activity of Na^+^ transporters could be a key factor in the development of hypertension during metabolic programming, but there have been no studies concerning the influence of protein restriction during lactation on the two ATP-dependent active Na^+^ transporters, (Na^+^+K^+^)ATPase and Na^+^-ATPase. These pumps are present in various nephron segments and particularly in the proximal tubule, a key structure responsible for approximately 70% of Na^+^ reabsorption. (Na^+^+K^+^)ATPase is considered to be responsible for the majority of Na^+^ reabsorption, and the ouabain-resistant Na^+^-ATPase is associated with the fine tuning of this process [17], [18]. Of particular interest are: (i) angiotensin II (Ang II), one of the main regulators of blood pressure and Na^+^ reabsorption [19], is a potent activator of the Na^+^-ATPase [20]; (ii) all components of the signal cascade that link Ang II receptors (AT_1_ and AT_2_ receptors) form a functional complex with the neighboring Na^+^-ATPase in basolateral membranes of kidney proximal tubule cells [20], [21], [22]; (iii) Overactive renal Na^+^-ATPase is one of the main molecular findings in obesity-associated hypertension [17] and in spontaneously hypertensive rats [23].

The aim of the present study was to examine whether metabolic programming during lactation affects the (Na^+^+K^+^)ATPase and Na^+^-ATPase activities of proximal tubule cells in young adult offspring from mothers that have suffered protein restriction during lactation. The results demonstrate that metabolic programming by undernutrition throughout lactation affects both Na^+^ transporters and key elements of the Ang II signaling pathways. At 60 days of age early alterations in glomerular morphometry, tissue collagen, glomerular filtration rate (GFR), protein excretion and urinary Na^+^ (U_Na_) accompany the modifications in Na^+^ pumping activity. These molecular, structural and functional modifications, leading to inappropriate Na^+^ handling in young adult rats, result in the development of arterial hypertension at a later age (150 days).

## Materials and Methods

### Rat groups

Experimental procedures involving rats were approved by the Committee for Ethics in Animal Experimentation of the Federal University of Rio de Janeiro (protocol N° IBCCF 104), and were performed in accordance with the Committee's guidelines.

Several series of successive breeding were performed as follows: Three-month old female rats (approximately 250 g) were caged with male rats in a 3∶1 ratio (controlled temperature of 23±3°C and 12 h light–dark cycle). After mating, each female was placed in an individual cage with free access to water and food until parturition. They were randomly separated into a control group (n = 4) with free access to water and a standard diet (20% protein), and a protein-restricted group (undernourished mothers; n = 4) with free access to water and a low-protein diet (8% protein) throughout lactation (0–21 days after parturition). Litters were adjusted to six males for each mother, generating two experimental groups: (i) control group (C, from control mothers); (ii) programmed group (P, from undernourished mothers). However, when the number of male pups was insufficient, female pups (no more than two) were included to maintain a litter size of six, but only male pups were studied after weaning; females were humanely sacrificed by decapitation. Four series of breeding with four rats (each group) provided sufficient offspring (a total of 16 pups in each group) to obtain the number of membranes required for the biochemical/immunochemical determinations. All series produced consistent results, with no differences being evident within the same class of experiment. Two series with two control and two undernourished females (eight pups in each group) were used for histological analysis. One series of breeding with three females produced litters (12 pups in each group) that were randomly divided into groups for blood pressure measurements and *in vivo* studies carried out in metabolic cages.

After weaning, the control and programmed groups were fed a commercial standard diet (Labina; 20% protein). Evolution of nutrition parameters was evaluated from weaning to 60 days of age, when animals were sacrificed for a first series of biochemical studies. Blood pressure was measured at this age and, in a parallel group, at 120 and 150 days of age to investigate the aforementioned possible late onset of hypertension.

### Diets, food and caloric intake, and body mass evolution

**Figure 1:**
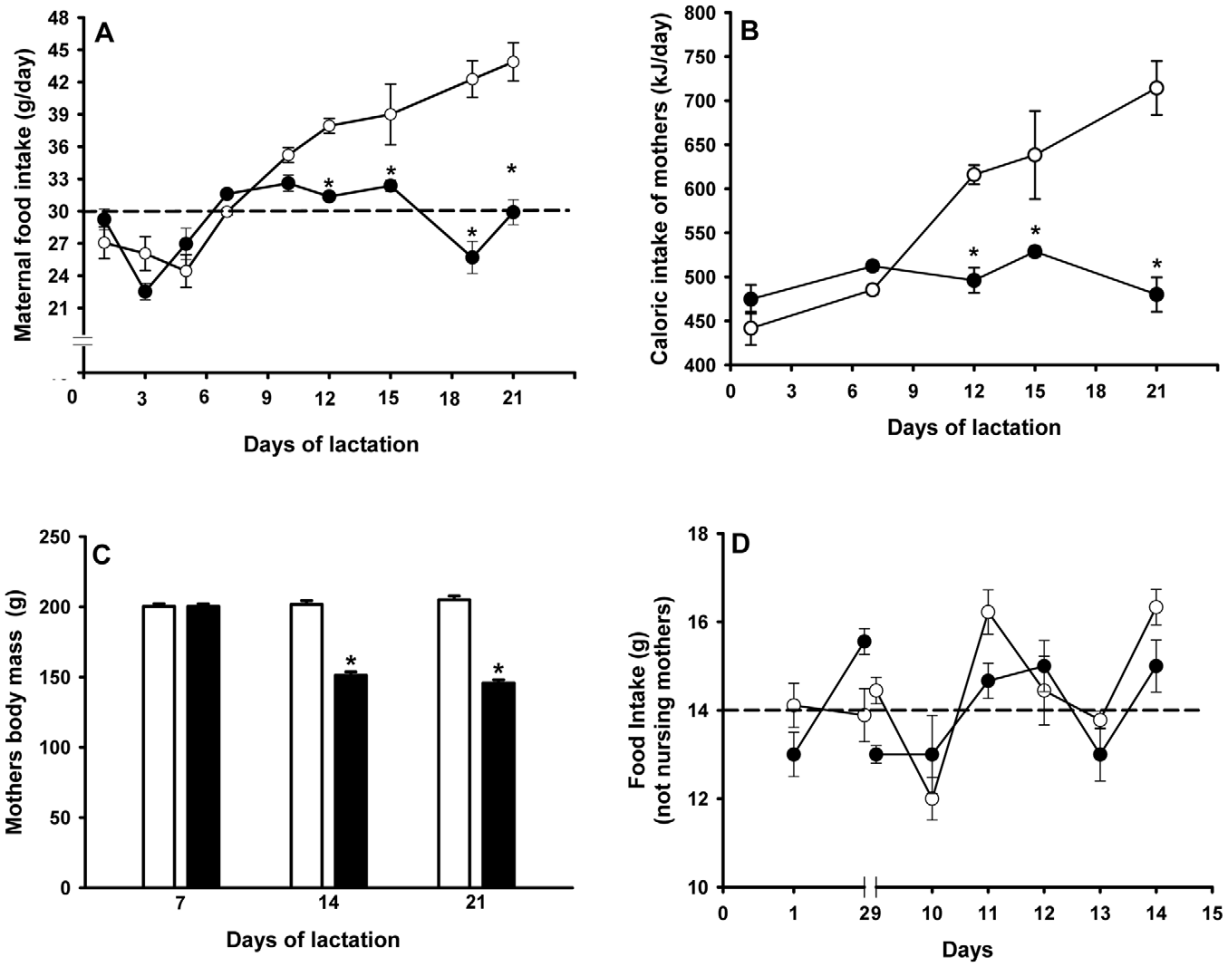
Maternal data: food and caloric intake, and body mass during lactation. (A) Mothers were given a control (open circles) or low-protein (filled circles) diet from parturition until the end of lactation. Diet compositions are described in Table 1. Dashed line indicates the mean value of chow ingestion by undernourished mothers during this period. Data are means ± S.E.M. (n = 10 in each group). (B) Maternal caloric intake (control, open circles; low-protein filled circles) during the indicated days of lactation calculated from: diet composition (Table 1), food intake (panel A), the caloric value of proteins (16.74×10^3^ kJ/kg), carbohydrates (16.74×10^3^ kJ/kg) and fat (37.66 kJ/kg), and a soybean oil average density of 926 kg/m^3^ according to the manufacturer. Data are means ± S.E.M. (n = 4 in each group). (C) Maternal body mass at the end of each week of lactation in control (empty bars) and low-protein-fed rats (filled bars). (D) Food intake by female rats (open circles for control; filled circles for low-protein) that were not nursing pups. Dashed line indicates the mean value of chow ingestion. In (A), (B) and (C): *statistically different from the corresponding control group (P<0.05 or less depending on the lactation day).

**Figure 2:**
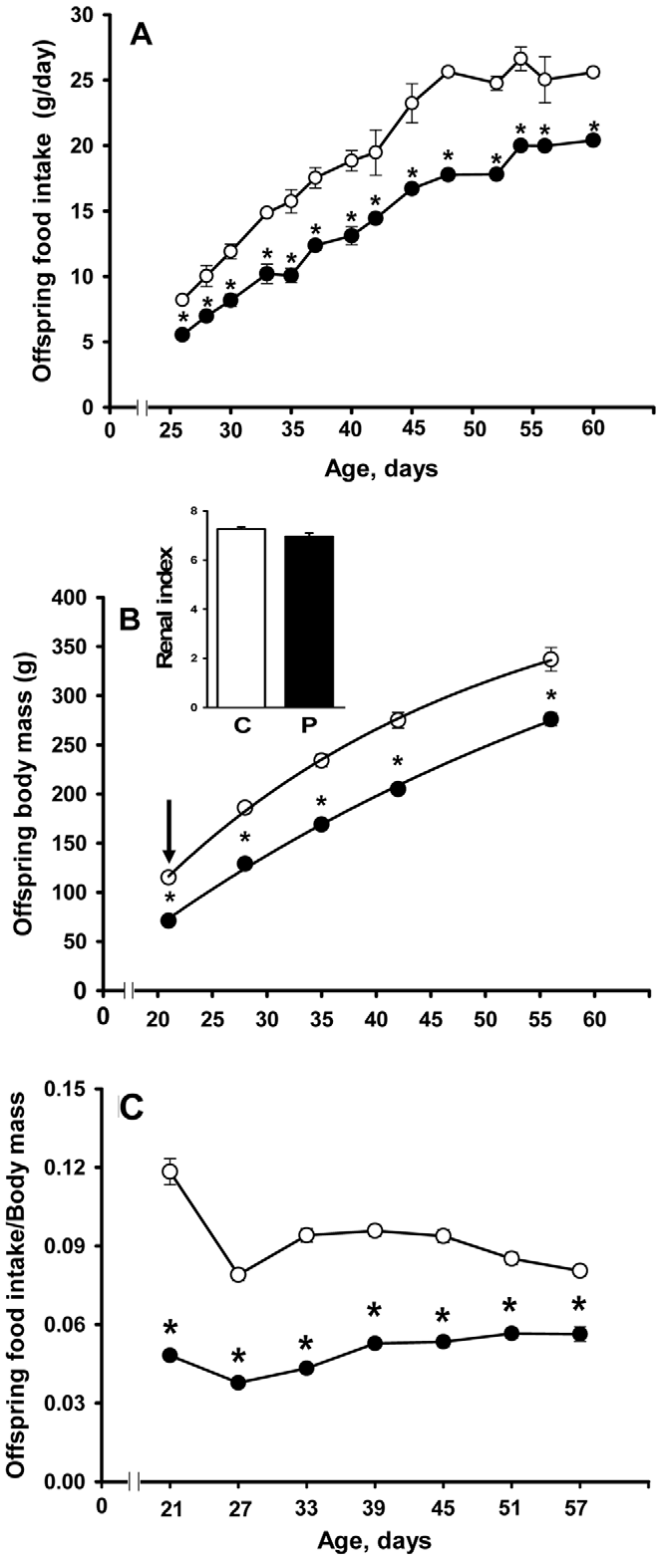
Food intake of offspring, evolution of progeny body mass after weaning, and relationship between body mass and food intake. zz(A) The offspring of control (open circles) and undernourished (filled circles) mothers were given a standard commercial diet from weaning to sacrifice. Data are means ± SEM (n = 14, control; n = 18, programmed). (B) The trajectories of growth in control (open circles) and programmed (filled circles) offspring were described by the function BM_t_ = BM_asy_ (1−e^−*kt*^), where BM_t_ is body mass at each indicated time *t*, BM_asy_ corresponds to the asymptotic value of the function and *k* is the first-order rate constant of growth. T_1/2_ was calculated by ln 2/*k*). Data points correspond to means ± SEM (n = 14, control; n = 18, programmed). The arrow indicates the significantly reduced body mass at weaning. Inset: kidney index calculated as the ratio between kidney mass and body mass (C: control; P: programmed). (C) Data points correspond to the ratio between the food intake at the indicated days (during 24 h) and body mass in control (open circles) and programmed (filled circles) offspring. In (A), (B) and (C): *statistically different from the corresponding control group (P<0.05 or less depending on the age).

The diets used during lactation were isoenergetic (Table 1), and were prepared in-house according to the AIN-93G recommendation for rodent diets [24]. Food and caloric intake of the control (normonourished) and undernourished female rats, and the evolution of maternal body mass, were evaluated throughout lactation as presented on the *abscissa* of Fig. 1. Control experiments concerning maternal food intake (both diets) were carried out using females of the same age that were not nursing pups. The body mass, kidney mass and food intake of the offspring were monitored from weaning until day 60 at the time intervals presented on the *abscissa* of Fig. 2.

**Table 1:** Composition of experimental diets.

Ingredient (g/kg)	Control (C)	Programmed (P)
Casein	200.0	80.0
L-cysteine	3.0	3.0
Corn starch	529.5	649.5
Sucrose	100.0	100.0
Cellulose	50.0	50.0
BHT^1^	0.014	0.014
Mineral mix^2^	35.0	35.0
Vitamin mix^2^	10.0	10.0
Choline bitartarate	2.5	2.5
Soybean oil^3^	70.0	70.0
Total energy in food (kJ/kg)	16,373	16,373

1 BHT: Tert-butylhydroquinone.

2 Vitamin and mineral mixtures were formulated according to the AIN-93G recommendation for rodent diets. More details of the composition are given in [23].

3 Soybean oil, ml/kg.

### Number of nephrons, glomerular morphometry and collagen density surface quantification

**Figure 3:**
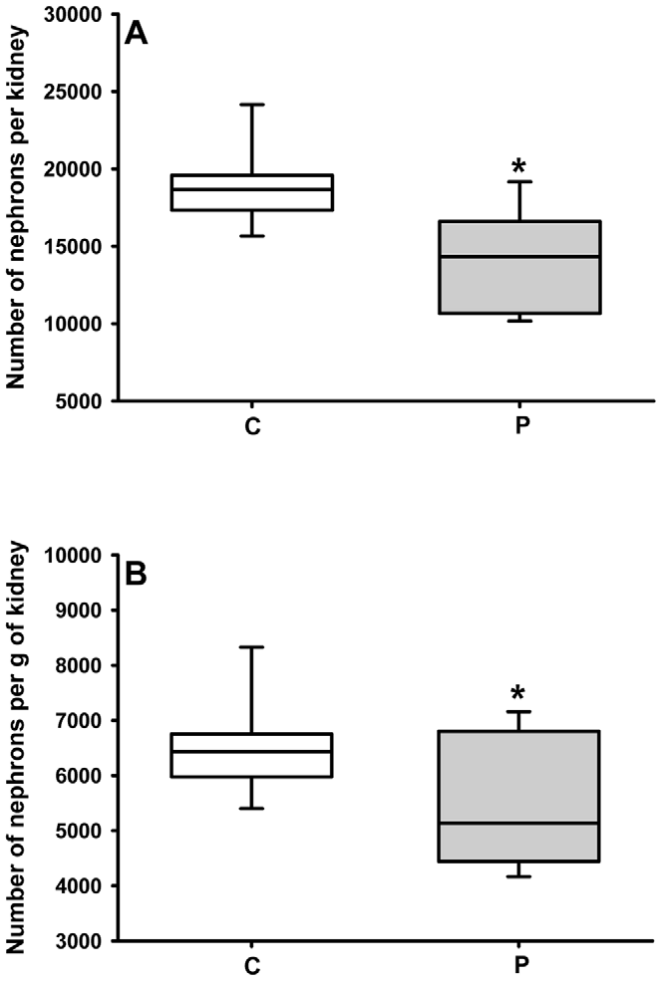
Number of nephrons in control and programmed offspring. After mechanical dissociation of the tubules, the number of glomeruli was evaluated by counting under light microscopy as described in the Materials and Methods. For the number of animals, number of homogenate samples, number of independent observations, total nephron count, intra- and inter-assay variability controls see Materials and Methods. (C: control; P: programmed). Statistical differences were assessed using the Mann-Whitney *U*-test. (A) Total number of nephrons per kidney; median = 18664 for control; median = 14331 for programmed; *U* = 88, *P<0.001. (B) Number of nephrons per g of kidney; median = 6436 for control; median = 5138 for programmed; *U* = 170.5, *P<0.01).

Four control rats and four programmed rats, aged 60 days, were used to determine the number of nephrons as described in [25], [26]. Briefly, one kidney from each animal was selected, weighed, sliced and incubated in hydrochloric acid 18.5% (w/v) for 2 h at room temperature. After gentle mechanical dissociation using a plastic syringe as a piston, the homogenate was adjusted to 10 ml with distilled water. Six 30 µl aliquots were obtained from each kidney homogenate and spread on glass slides over a surface of 4.5 cm^2^. Two observers (double blind counting) counted glomeruli in every aliquot using a light microscope and in each group the number was 24. A total of 1324 nephrons were counted in the control rats and 975 in programmed animals. Statistical controls for intra-assay variability during counting within each group produced a P value≥0.1; inter-assay variability analysis of the method between control and programmed rats produced a P value≤0.01 (see statistical analysis below and Fig. 3).

Three control and three programmed rats, aged 60 days, were used for morphometric studies. One kidney from each animal were mid-frontally sectioned into two pieces, fixed with formaldehyde (10% w/v in 10 mM phosphate buffer pH 7.2) for three days, dehydrated in rising concentrations of ethanol with successive 30 min immersion periods, and embedded in paraffin. Four cortical sections, 4 µm thick and avoiding the juxtamedullar boundary, were randomly chosen and stained with hematoxylin-eosin (HE), and the surface areas of the glomerular components were determined using the software Image-Pro Plus (Media Cybernetics) connected to a light microscope. Five images (2048×1536 pixels) were captured from each section and each of these was screened. A total of 60 micrographs per group were screened; between 20−22 glomeruli were quantified per kidney using a 200× objective lens. For statistical analysis the screening was carried out in triplicate.

To evaluate collagen density (% of the total surface in the captured images), Picro Sirius red stained sections were obtained as described for morphometric studies and elsewhere [27], with the exception that micrographs were separately captured from cortical and medullary regions. Twenty photomicrographs from each section were evaluated using a 200× objective lens. The areas were randomly chosen, although fields containing medium-sized blood vessels were avoided.

### Isolation of proximal tubule cell membranes

Rats from various litters were sacrificed by decapitation and the kidneys were collected and maintained in cold isotonic buffer containing 250 mM sucrose, 10 mM Hepes-Tris (pH 7.4), 2 mM EDTA and 0.15 mg/ml trypsin inhibitor type II-S (Sigma-Aldrich). The membranes were prepared according to [28] from the outer cortex (*cortex corticis*) of the kidney, in which more than 90% of the cell population corresponded to proximal tubules [29], [30]. Briefly, thin transverse slices of the *cortex corticis* (0.5 mm) were removed with a Stadie-Riggs microtome and carefully dissected with scissors to eliminate contamination from other tissues. The fragments were homogenized in 4 ml per gram of the cold solution described above. The homogenate was centrifuged at 755× *g* for 15 min to remove cell debris and nuclei, and the resulting supernatant was centrifuged at 8,500× *g* for 20 min, followed by ultracentrifugation at 35,000× *g* for 45 min. The final pellet was resuspended in 250 mM sucrose and stored at −20°C. Protein content was determined using the Folin phenol method [31]. Controls for enrichment with basolateral membranes (3–4 fold with respect to the total homogenate) and for minimal residual contamination with intracellular membranes and cytosol were as in [28]. The preparation preserves apical membranes. No attempt at further enrichment was made in this case as the ATP-driven Na^+^ transporters are exclusively located in basolateral membranes, and a low yield of purified basolateral membranes was obtained using the Percoll gradient method with the minimum number of animals recommended by the Committee for Ethics in Animal Experimentation.

### Measurement of ATPase activities

The (Na^+^+K^+^)ATPase and the furosemide-sensitive (ouabain-insensitive) Na^+^-ATPase activities were measured *via* P_i_ [32] or ^32^P_i_ [18] released from unlabeled ATP or [γ-^32^P]ATP, respectively. Routine controls had identical values for ATPase activities irrespective of which method was used. In (Na^+^+K^+^)ATPase assays, the membranes (0.05 mg/ml, final concentration) were pre-incubated at 37°C for 10 min with 50 mM Bis-Tris-propane (pH 7.4), 0.2 mM EDTA, 5 mM MgCl_2_ and 120 mM NaCl in the absence or presence of 2 mM ouabain. The reaction was started by adding 5 mM ATP and 24 mM KCl (final concentrations). The reaction was stopped after 10 min by adding two vols of 0.1 M HCl-activated charcoal. P_i_ was measured spectrophotometrically in an aliquot of the supernatant obtained after centrifugation of the charcoal suspension at 1500× *g* for 5 min. The (Na^+^+K^+^)ATPase activity was calculated as the difference between the P_i_ released in the absence and presence of ouabain. The K^+^ and Na^+^ concentrations were changed in some experiments and the concentrations used are presented on the *abscissa* of the corresponding figure.

For the ouabain-insensitive Na^+^-ATPase assays, the membranes (0.2 mg/ml final concentration) were pre-incubated with 2 mM ouabain in the presence of 20 mM Hepes-Tris (pH 7.0), 10 mM MgCl_2_ and 120 mM NaCl without or with 2 mM furosemide. The hydrolysis reaction was started by adding [γ-^32^P]ATP (5 mM, specific activity ∼2 MBq/µmol) and stopped after 10 min by adding two vols 0.1 M HCl-activated charcoal. The ouabain-insensitive Na^+^-ATPase activity was calculated from the difference between levels of ^32^P_i_ released in the absence and presence of furosemide. Released ^32^P_i_ was quantified using liquid scintillation counting in an aliquot of the supernatant obtained after centrifugation of the charcoal suspension (1500× *g* for 5 min) [18].

### Protein kinase activities

The activities of calphostin-sensitive protein kinase C (PKC) and cAMP-dependent protein kinase (PKA) associated with the isolated membranes were assessed by measuring the incorporation of the γ-phosphoryl group of [γ-^32^P]ATP into histone in the absence and presence of calphostin (PKC assays) or in the absence and presence of the PKA specific inhibitor, the peptide PKAi_(5–24)_ [33] (PKA assays), as recently described [18].

### SDS-PAGE and Western blotting of (Na^+^+K^+^)ATPase, Ang II receptors and membrane-associated protein kinases

The (Na^+^+K^+^)ATPase α_1_-catalytic subunit was immunodetected in isolated membranes using a goat polyclonal antibody against this subunit (α1[N-15], Sigma-Aldrich) and an anti-goat secondary antibody (Santa Cruz Biotechnology). AT_1_ and AT_2_ receptors, the calphostin-sensitive α and ε PKC isoforms and the α-catalytic subunit of PKA were immunodetected in isolated membranes using the corresponding polyclonal antibodies (Santa Cruz Biotechnology), as previously described [18], [34]; β-actin was probed with a monoclonal antibody (Sigma-Aldrich) after Ang II receptors had been immunoprecipitated [34]. The proteins were separated in a 10% gel using SDS PAGE and transferred to nitrocellulose membranes at 350 mA. Non-specific binding was prevented by incubating the membranes with 5% non-fat milk in Tris buffered saline (TBS, pH 7.6) for 1 h. The membranes were probed with the corresponding primary antibodies (1∶500 dilutions) for 1 h at room temperature with stirring, washed three times with TBS containing 0.1% Tween 20 (TBST), incubated with the secondary antibody, washed and visualized with ECL™ (GE Healthcare). The gels were stained with Ponceau Red to evaluate the levels of protein in each band to normalize levels of expression by the protein load. Preliminary experimental controls demonstrated that Ponceau red correlates better (r = 0.998) than β-actin (r = 0.948) with the theoretical protein loading. However, there was a very good correlation between the Ponceau Red intensity and the β-actin signal, using the same membrane for detection (r = 0.964). Therefore, Ponceau red or β-actin were used to assess loading, as both provided the same results. The band intensities were quantified using Scion Image software.

### Measurement of blood pressure

Measurements were carried out as in [35]. Briefly, rats were anesthetized with an intraperitoneal injection of a mixture of ketamine (100 mg/kg) and xylazine (50 mg/kg). A double catheter (PE-50 into PE-10; Clay Adams) was inserted in the right femoral artery and connected to a pressure transducer (TSD 104A, Biopac Systems). Recording was carried out using the Biopac System MP 100 (precision 1 mmHg) and the software was provided by the same manufacturer.

### *In vivo* studies using metabolic cages

The glomerular filtration rate (GFR), urinary Na^+^ excretion (U_Na_) and proteinuria were evaluated using metabolic cages (Instrulab) in offspring aged 60 days. The animals were placed in cages for 24 h in ambient conditions as described above. The GFR was measured by determining creatinine clearance using a kit (Analisa) for the spectrophotometric alkaline pycrate method. A colorimetric kit (Human Diagnostics Worldwide) was used to measure U_Na_, and proteinuria was determined using the Folin reagent method [31].

### Statistical analysis

The data are presented as means ± S.E.M. Differences between groups were analyzed using an unpaired Student's *t*-test and one-way ANOVA followed by a Tukey test, or two-way ANOVA followed by a Bonferroni test, where required. The Mann-Whitney *U*-test was used to assess differences in number of glomeruli between the two groups and controls for intra- and inter-assay variability. In all cases the differences were considered significant when P<0.05. Where the results were expressed as a percentage of the control group, the S.E.M. was calculated from the absolute data and transformed to percentages, and statistical analysis using parametric tests was carried out for absolute values. Furthermore, the Mann-Whitney *U*-test was used to compare percentage values.

## Results

### Food intake of mothers during lactation and of progeny after weaning

Mothers that received the balanced control diet gradually increased their food intake from day five after parturition to weaning, presumably to adjust to the nutritional challenges of lactation (Fig. 1A). In contrast, food intake in undernourished mothers remained essentially unchanged throughout this period, with a mean value of 29 g/day (dashed line in Fig. 1A). Therefore, a significant difference in food intake was evident from day 12 to weaning. Both diets were isoenergetic (16373 kJ/kg). Therefore, the profile of caloric intake follows the food consumption profile (Fig. 1B). As a consequence of the constancy of the alimentary profile, as the energetic demand increased during lactation, mothers receiving the protein deficient diet exhibited a significant decrease in their body mass at 14 and 21 days (Fig. 1C), *i.e.* this was synchronous with the establishment of a difference in the food/energy intake. Food intake by mothers subjected to the normal and low-protein diet was similar when they were not nursing pups (Fig. 1D). Differences in alimentary behavior between control and undernourished mothers that were encountered from the 10^th^ day of lactation (Fig. 1A) were no longer evident, with lower food intake probably reflecting the lower nutritional demand compared with breastfeeding mothers.

Programmed offspring had a significantly lower food intake than the control group, and this alimentary profile persisted from weaning until day 60 (5.5±0.2 vs. 8.2±0.2 at day five after weaning; 20.4±0.2 vs. 25.6±0.3 at day 60; P<0.05) (Fig. 2A).

### Growth trajectories in offspring and the relationship to food intake

The growth curve of programmed rats demonstrated a significant decrease in body mass from weaning to 60 days of age (Fig. 2B). The rate constant of growth, *k*, decreased from 0.0357±0.0034 days^−1^ in the control group to 0.0204±0.0014 days^−1^ in programmed offspring. Therefore, the T_1/2_ of growth of the programmed group extrapolated into adult life increased (36.8±2.5 *vs.* 20.7±1.4 days in controls). The offspring food intake/body mass ratio at different days of age (Fig. 2C) was lower in the programmed group. Renal mass accompanied the decrease in body mass, though the renal index (kidney mass/body mass) remained unchanged (inset to Fig. 2B).

### Number of nephrons, glomerular morphometry and collagen deposition

Fig. 3 demonstrates that programming during lactation led to a decreased number of nephrons in the offspring. Differences in the total number of nephrons (Fig. 3A) were not due to decreased kidney size alone: factoring for kidney mass did not abolish these differences (Fig. 3B). Although the impact on renal molecular machinery of the progeny is different depending on the period of maternal undernutrion (see Discussion below), the results depicted in Fig. 3 are comparable to those in a model of protein restriction during pregnancy [26].

**Figure 4:**
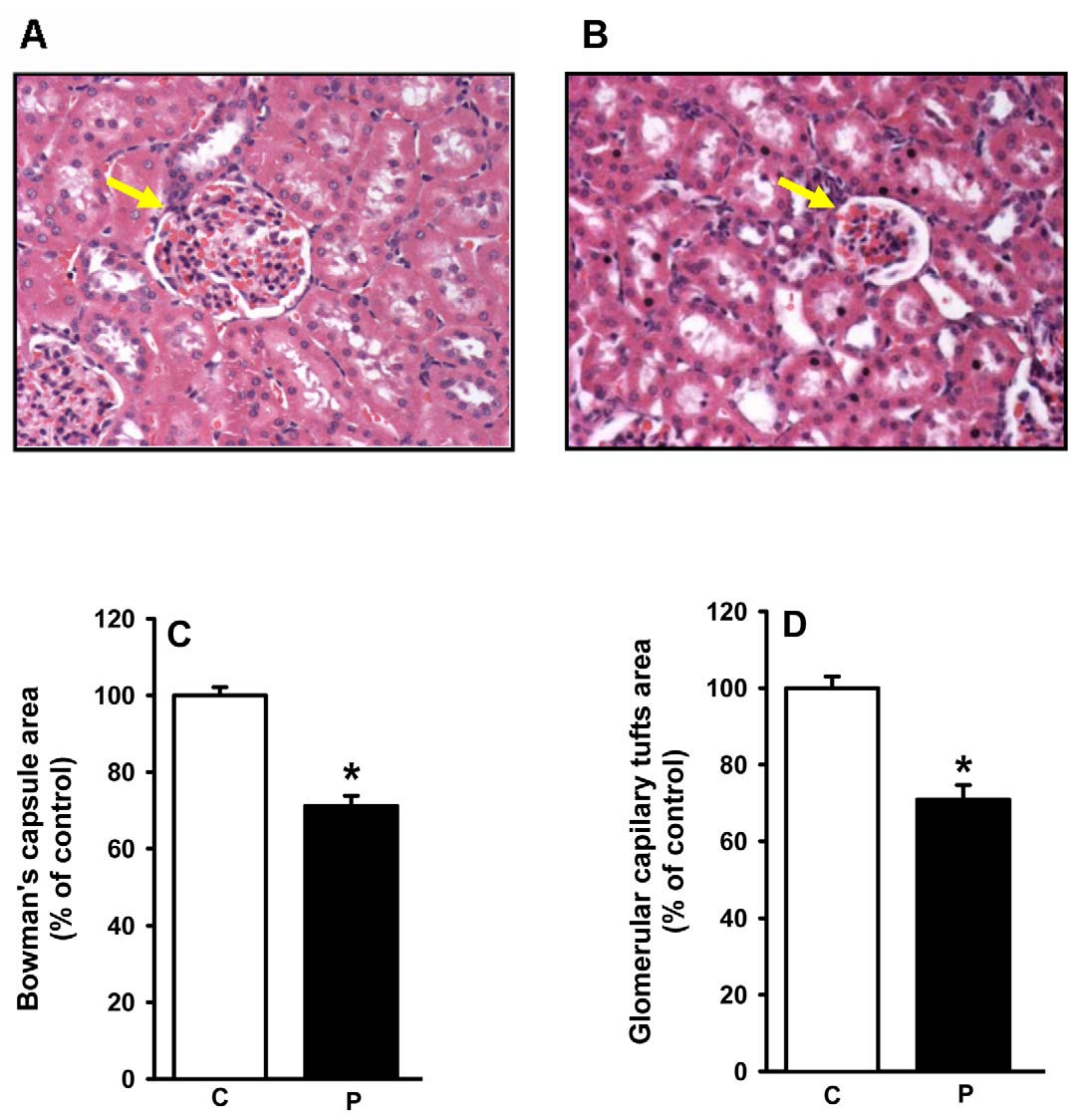
Glomerular morphometry in control and programmed offspring. The areas of the Bowman's capsule and of the capillary tuft in the cross section of glomeruli were assessed as described in the Materials and Methods. For number of rats, number of screened tissue sections and number of glomeruli counted, see Materials and Methods. Panels A and B show representative photomicrographs (200×) of HE-stained sagital kidney sections of control and programmed rats, respectively. Arrows indicate glomerular structures. Panel C: quantification of the Bowman's capsule area expressed as a percentage of the control group. Results were statistically analyzed and then converted to percentage values (n = 3; *P<0.001). Panel D: quantification of the capillary tuft area expressed as a percentage of the control group (n = 3; *P<0.001). On the *abscissae* of panels (C) and (D), the capital letters C and P indicate the control and programmed groups, respectively.

**Figure 5:**
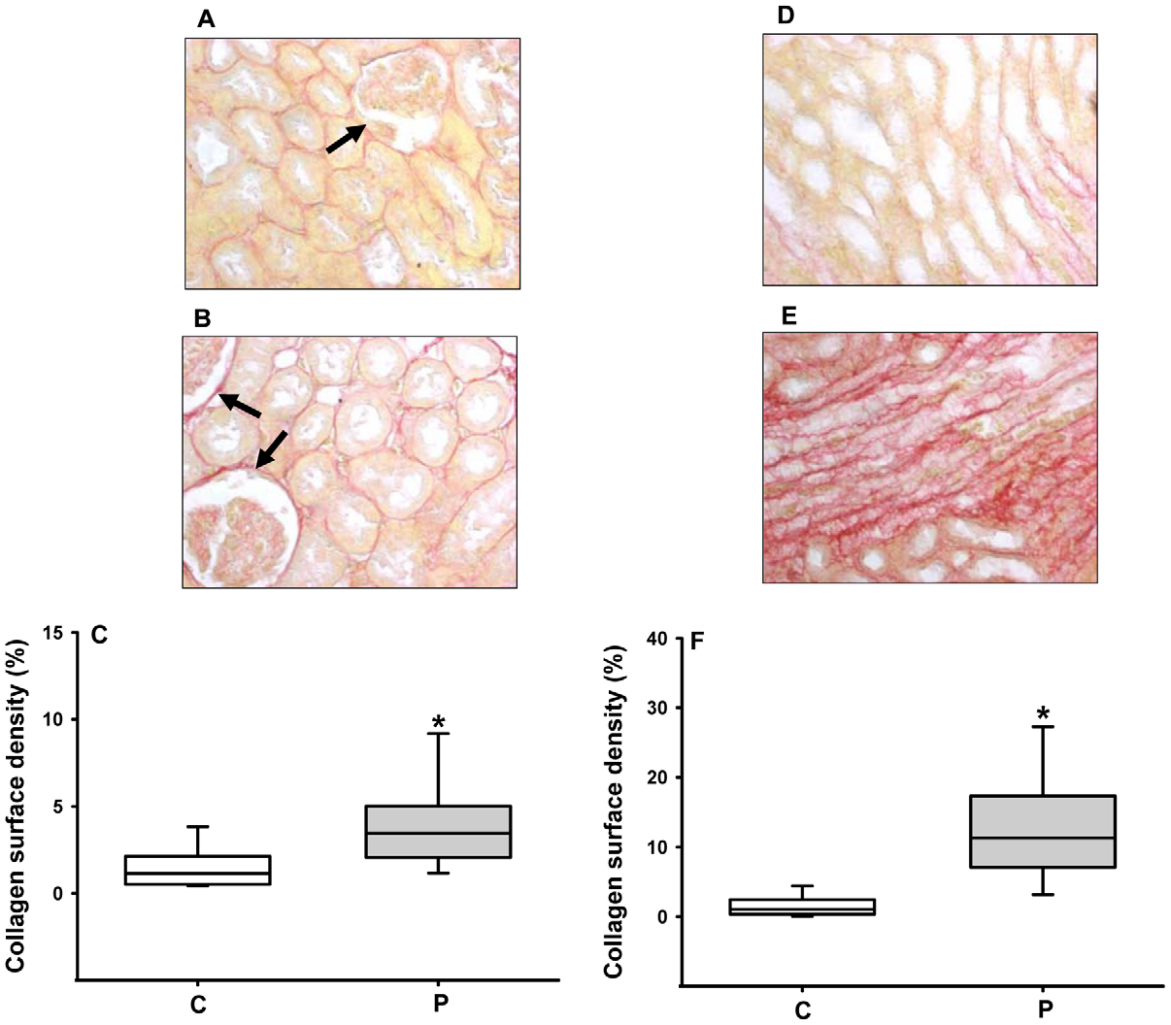
Collagen deposition in the cortical and medullary regions. Collagen surface density was quantified as described in the Materials and Methods (n = 3 for each group). Panels A and B present representative Picro Sirius-stained sagital cortex sections of control and programmed rats, respectively. Panel C: graphic representation of collagen surface density in the cortex. Arrows indicate pericapsular collagen deposits and, in the case of panel B, a large-size glomerulus in the predominat population of glomeruli with decreased volume. Panels D and E present representative medullary images from control and programmed rats, respectively. Panel F: graphic representation of collagen surface density in the medulla. Statistical differences were assayed using the Mann-Whitney *U*-test. On the *abscissae* of panels (C) and (F), the capital letters C and P indicate the control and programmed groups, respectively. In (C) median for control = 1.14; median for programmed = 3.44; *U* = 179; P<0.0001. In (F) median for control = 1.05; median for programmed = 11.32; *U* = 49; P<0.0001).

The reduced number of nephrons was accompanied by a reduction of the total area of the Bowman's capsule (Fig. 4A and 4C) and the surface area corresponding to the glomerular capillary tuft (Fig. 4B and 4D). Intense diffuse collagen deposition was present in the cortex and medulla of programmed animals (Fig. 5); of particular interest was increased collagen density in glomeruli (compare images pointed by arrows in Fig. 5A and 5B).

### Glomerular filtration rate, Na^+^ excretion and proteinuria

A reduced number of glomeruli could result in hyperfiltration in the remaining nephrons, with increased filtered Na^+^ load and modifications in renal Na^+^ handling [12]. Therefore, *in vivo* studies were carried out in metabolic cages with rats aged 60 days to compare GFR and Na^+^ excretion (U_Na_) between the two offspring groups. Table 2 demonstrates that these renal parameters were significantly increased in the programmed group. Evaluation of urinary excretion of total protein demonstrated an intense proteinuria in the programmed group (Table 2).

**Table 2:** Renal function parameters^1^.

	Control (C)	Programmed (P)
GFR, µl/min/100 g BM	254.1±26.1	427.8±16.2*
U_Na_, mmol/100 g BM/24 h	0.5±0.1	0.9±0.1*
Proteinuria, mg/100 g BM/24 h	2.6±0.4	11.5±0.6*

1 GFR, Glomerular filtration rate; U_Na_, Urinary excretion of Na^+^; BM, body mass;

*P<0.001.

### (Na^+^+K^+^)ATPase activity and expression of its α_1_-subunit in proximal tubules from programmed rats

**Figure 6:**
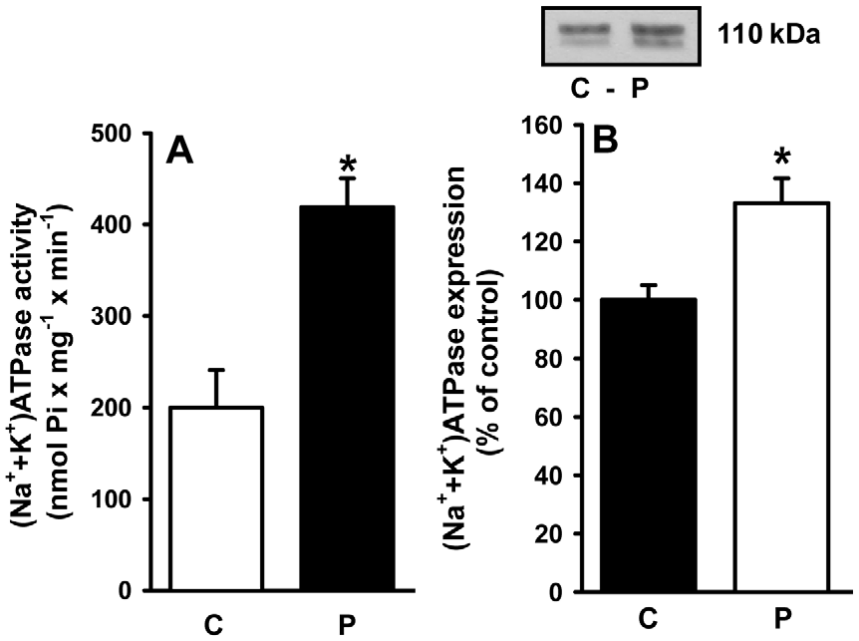
Activity and expression of (Na^+^+K^+^)ATPase in basolateral membranes of kidney proximal tubule cells. (A) (Na^+^+K^+^)ATPase activity was measured in membranes isolated from control (C) and programmed (P) offspring, as indicated on the *abscissa*. Data are means ± S.E.M. of six (control) and eight (programmed) determinations carried out in triplicate using different membrane (rat) preparations. *Statistically different from C (P<0.01). (B) Expression of (Na^+^+K^+^)ATPase. Upper panel: representative immunoblotting of α_1_ subunit. Lower panel: densitometric quantification (means ± S.E.M.) of five simultaneous determinations carried out with four different membrane preparations from each group (C and P). The band intensity of the control group was taken as 100%. *Statistically different from the control group (P<0.05).

**Figure 7:**
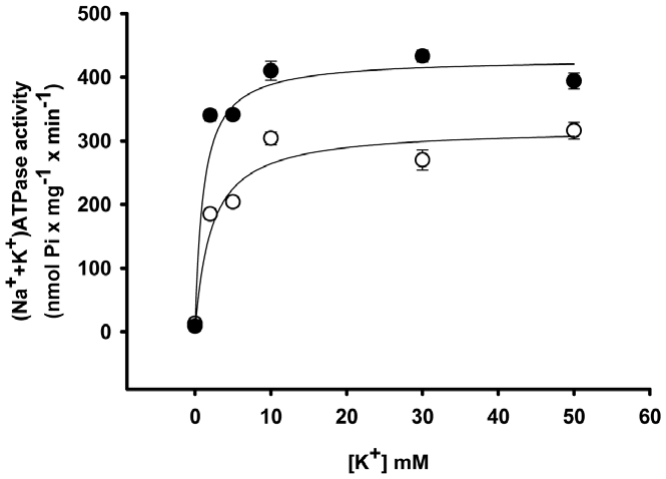
Apparent affinity for K^+^ of renal (Na^+^+K^+^)ATPase. The (Na^+^+K^+^)ATPase activity was assayed at the K^+^ concentrations presented on the *abscissa* (starting with a contaminant concentration of 0.1 mM K^+^ according to flame photometric determinations performed after preparation of the solution was completed). The Na^+^ concentrations ranged from 150 to 100 mM to keep the sum of Na^+^ plus K^+^ concentrations equal to 150 mM in all tubes. The hyperbolic function *v* = V_max_×[K^+^]/(K_0.5_+[K^+^]) was adjusted to the experimental points obtained with the use of membranes isolated from control (open circles) and programmed (filled circles) offspring. Data are means ± S.E.M. of at least seven determinations carried out in triplicate with four different preparations from each group (C and P). The abbreviations of the expression correspond to activity at each K^+^ concentration (*v*), extrapolated maximal velocity (V_max_) and to the K^+^ concentration at which *v* = V_max_/2 (K_0.5_).

Maternal protein deficiency throughout lactation programmed a significant increase in (Na^+^+K^+^)ATPase activity (more than 100%) (Fig. 6A), with a 35% increase in the expression of the housekeeping α_1_-subunit of the pump (Fig. 6B). The increases in activity and protein expression were not similar, suggesting that the ATPase could undergo molecular modifications. In fact, the K_0.5_ for K^+^ during the catalytic cycle decreased from 1.7 mM to 0.5 mM (Fig. 7) whereas the affinity for Na^+^ was not significantly altered (data not shown). With a membrane preparation lacking the cytosolic components required for the modulation of (Na^+^+K^+^)ATPase in proximal tubules [36], there was no response of (Na^+^+K^+^)ATPase to Ang II in either group using a wide concentration range (10^−14^ to 10^−6^ M; data not shown).

### Influence of programming on the ouabain-insensitive Na^+^-ATPase and on its response to Ang II

**Figure 8:**
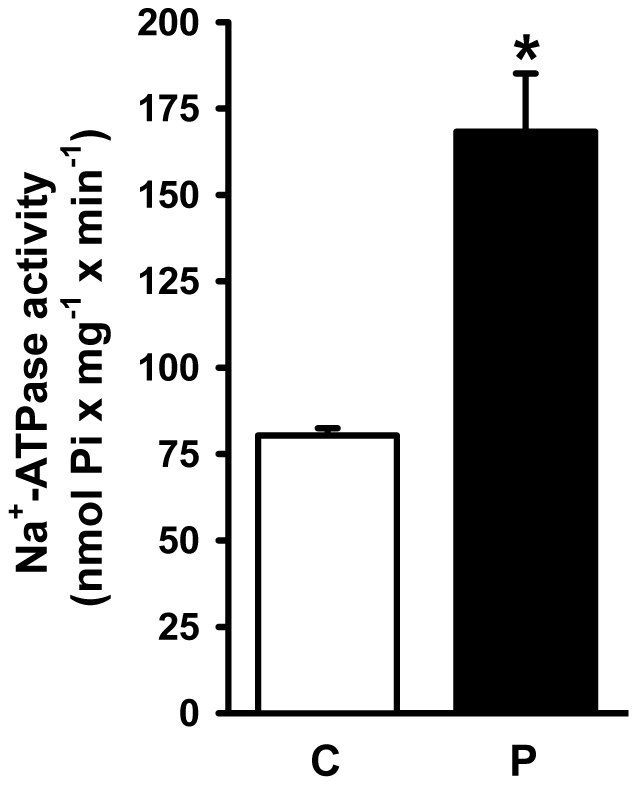
Ouabain-insensitive Na^+^-ATPase activity. The Na^+^-ATPase activity was assayed in membranes isolated from control (C) and programmed (P) offspring, as indicated on the *abscissa*. Data are means ± S.E.M. of 11 determinations (both groups) carried out in triplicate using four different membrane preparations from each group (C and P). *Statistically different from the control group (P<0.001).

**Figure 9:**
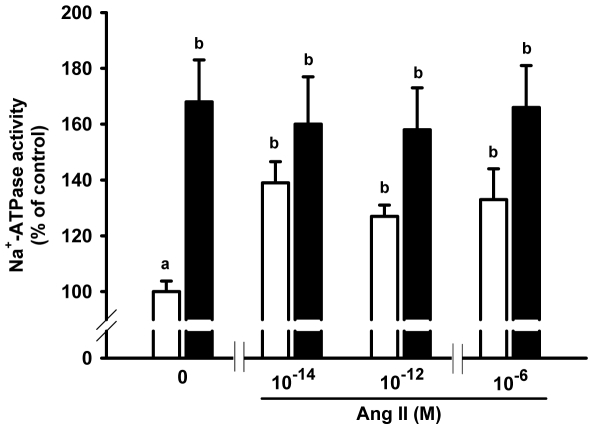
Response of the ouabain-insensitive Na^+^-ATPase to Ang II. Na^+^-ATPase activity from basolateral membranes of control (open bars) and programmed (filled bars) offspring was assayed in the absence or presence of Ang II at the concentrations presented on the *abscissa*. Mean activity values and standard errors were calculated from the absolute values and expressed as percentages (see Statistical Analysis sub-section). Na^+^-ATPase activity of control with no Ang II was taken as 100%. Different lower-case letters indicate statistical difference (P<0.05; one-way ANOVA to evaluate Ang II effects within each group and two-way ANOVA followed by Bonferroni test for the comparison of Ang II effects between control and programmed groups). Determinations were carried out in triplicate using different preparations (n = 5 for each group at each Ang II concentration).

Na^+^-ATPase activity increased in the adult progeny of rats that had been undernourished during lactation (Fig. 8), indicating that the two modes of transepithelial Na^+^ transport were programmed in their offspring. The Na^+^-ATPase of the programmed rats lost its physiological response to Ang II (10^−14^ M), and this lack of response was sustained at higher concentrations (Fig. 9). However, the Na^+^-ATPase of the programmed group behaved as if it were constitutively activated by Ang II, as there was no difference between the activities without Ang II and the control with Ang II (columns marked with the same lowercase letters in Fig. 9).

### Alterations in the expression of Ang II receptors in adult progeny of malnourished mothers

**Figure 10:**
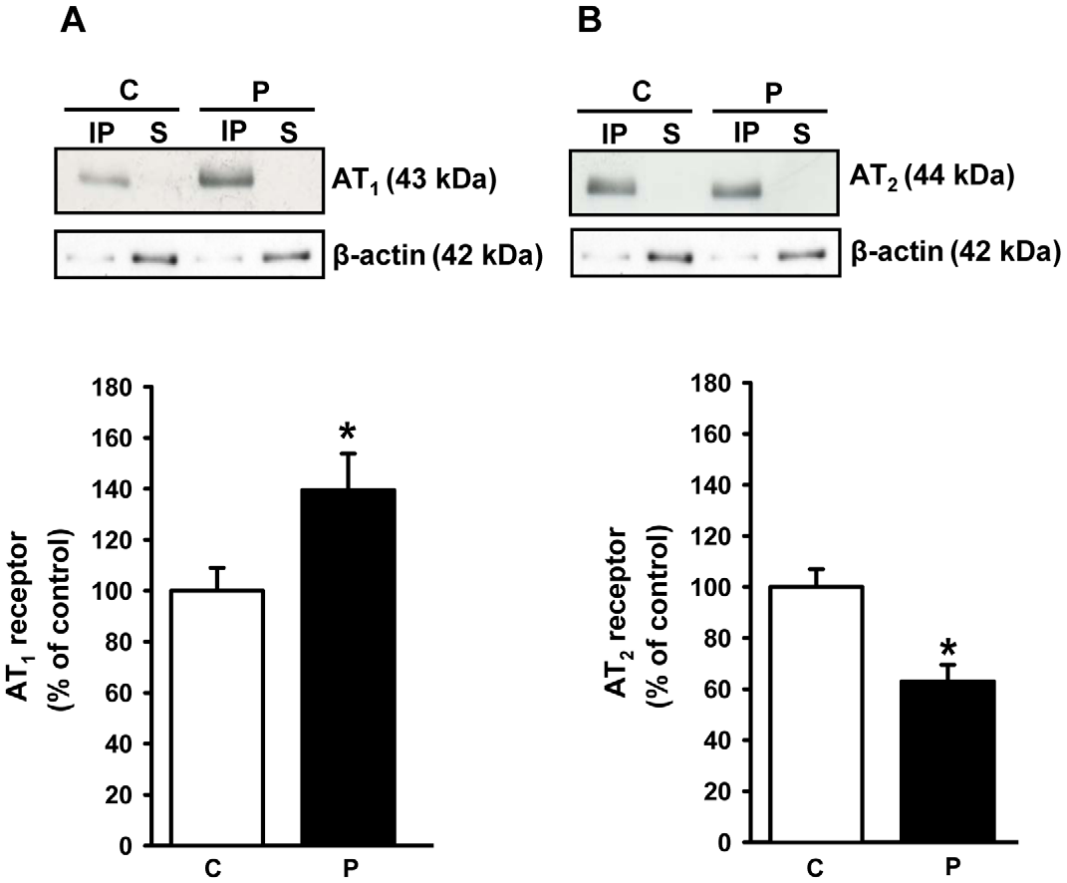
Expression of Ang II receptors. Ang II receptors (AT_1_ and AT_2_) were immunodetected in proximal tubule cell membranes of control (C) and programmed (P) offspring. (A) AT_1_ receptors. (B) AT_2_ receptors. In (A) and (B): upper panels show representative immunoblots after immunoprecipitation with the corresponding antibodies (IP, immunoprecitate; S, supernatant); middle panels show immunodetection of β-actin probed in the same membrane, which was used to asses protein loading in the gels; lower panels show densitometric quantification (means ± S.E.M.) of five immunodetections carried out with different membrane preparations. The band intensity of the respective control group was taken as 100%. *Statistically different from the control group (P<0.05).

Programming altered other components of the Ang II pathways in renal membranes. AT_1_ receptor expression increased (Fig. 10A), whereas AT_2_ receptor expression decreased (Fig. 10B) reciprocally.

### Membrane-associated PKC: activity and expression

**Figure 11:**
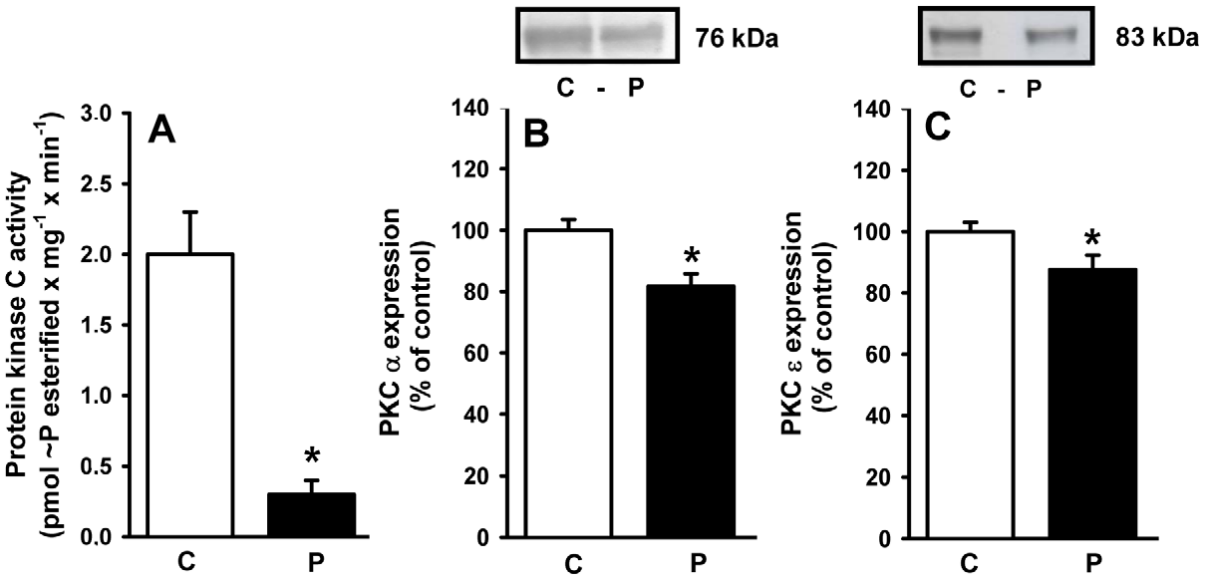
Activity and expression of calphostin-sensitive protein kinase C (PKC) in membranes from proximal tubule cells of control (C) and programmed (P) offspring. (A) PKC activity measured in six different membrane (rats) preparations. The results are means ± S.E.M. *Statistically different from C (P<0.001). (B) and (C) Immunodetection of the calphostin-sensitive α and ε isoforms of PKC, respectively. Upper panels: representative immunoblottings. Lower panels: densitometric quantification (means ± S.E.M.) of five immunodetections carried out with different membrane preparations. The band intensity of the respective control group was taken as 100%. *Statistically different from the control group (P<0.05).

PKC is a key intermediate in the signaling cascade that links AT_1_ receptors to the Na^+^-ATPase in kidney proximal tubule cells [20]. The adult programmed offspring had a membrane-associated PKC activity that was 15% of that in the control group (Fig. 11A). However, immunodetection experiments demonstrated decreases of 20% and 13% in expression of the calphostin-sensitive α and ε isoforms of PKC, respectively (Fig. 11B and 11C), but these decreases were statistically significant.

### Membrane-associated PKA: activity and expression

**Figure 12:**
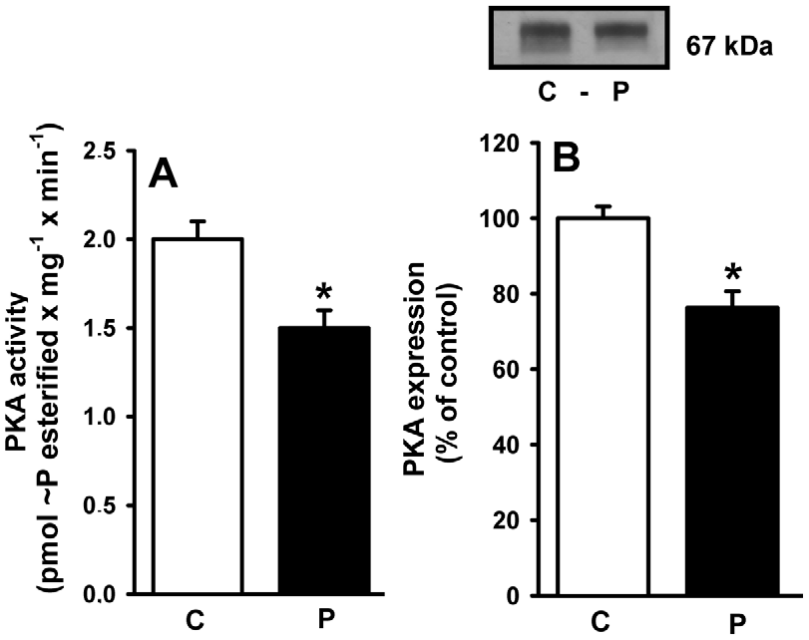
Activity and expression of cAMP-dependent protein kinase (PKA) in membranes from proximal tubule cells of control (C) and programmed (P) offspring. (A) PKA activity measured in six different membrane (rats) preparations. The results are means ± S.E.M. *Statistically different from the control group (P<0.05). (B) Immunodetection of PKA α-catalytic subunit. Upper panel: representative immunoblotting. Lower panel: densitometric quantification (means ± S.E.M.) of five immunodetections carried out with different membrane preparations. The band intensity of the respective control group was taken as 100%. *Statistically different from the control group (P<0.05).

This kinase has been implicated as an antagonist of PKC in its actions on the renal Na^+^-ATPase [37], [38]. In contrast to the PKC findings, the activity (Fig. 12A) and expression (Fig. 12B) of PKA in the membranes of malnourished rats decreased to comparable levels.

### Blood pressure in programmed rats

**Figure 13:**
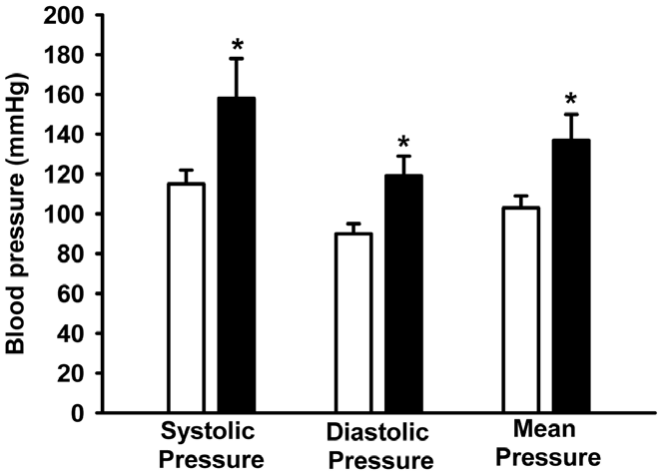
Later increase in arterial blood pressure in programmed offspring. Blood pressure was recorded in rats aged 150 days as described in the Materials and Methods (control, empty bars; programmed, filled bars). Data are means ± S.E.M. (n = 5 in both groups). *Statiscally different from the control group (P<0.05).

Programmed rats had normal arterial blood pressure at 60 and 120 days of age (data not shown). However, late onset hypertension occurred in the programmed progeny. Fig. 13 demonstrates a significant increase in the systolic and diastolic pressure of programmed rats aged 150 days.

## Discussion

The present work focused on the influence of maternal protein restriction during lactation on the ATP-dependent renal Na^+^ transporters in offspring. This narrow period of pup development is considered one of the most important for the development of metabolic programming [7], [8], and nephrogenesis is completed during this period [39], [40]. Fig. 1 demonstrates that despite the increased demand of lactation from an equal number of pups, the alimentary rhythm of undernourished mothers entailed two restrictions, low protein content of the diet and lower total food intake with decreased available energy, which could cause metabolic programming in the offspring. The abnormal alimentary habit of undernourished mothers during lactation is probably due to low protein-associated hyperleptinemia [41], [42], [43], [44], which could lead to satiety despite an increased energy demand as lactation ensued. The accompanying hypoprolactinemia contributed to the possible anorexigenic hormonal balance due to the orexigenic actions of prolactin [45]. The reduced body mass of the offspring at weaning (more than 40%; arrow in Fig. 2B) was a result of the low-protein induced lactogenesis failure [44].

These maternal alimentary restrictions could contribute to the imprinting of metabolic programming of the progeny as they consistently consumed less of the normal diet despite it being offered ad libitum (Fig. 2A). This decreased food intake led to a reduced body mass throughout the growth period analyzed in the present study (Fig. 2B). Since the offspring food intake/body mass ratio after weaning remained permanently lower (Fig. 2C), it is likely that the reduction in food intake was not due to lower body mass but because the rats were programmed to have this alimentary behavior having had reduced energy availability from their undernourished mothers (Fig. 1A and 1B). As discussed above, in a similar model of maternal undernutrition [44], plasma levels of the anorexigenic leptin were increased and levels of the orexigenic prolactin were decreased. In addition to decreasing maternal food intake, this hormone imbalance could have imprinted an impaired dietary behavior on the progeny. Evidence for this concerns elevated plasma levels of leptin in pups from mothers that had been undernourished during lactation, as a result of direct transmission via milk [46].

Acid maceration is not the gold-standard method for estimating the number of nephrons. However, reproducibility of counting using different kidneys and more than one independent observer, together with comparable control values with data from other laboratories [25], [26], demonstrated a profound influence of programming in terms of the number of nephrons in adult kidneys. The reduced number of nephrons (Fig. 3) and the structural glomerular alterations (Fig. 4) demonstrate that the decreased kidney mass of programmed animals was not a simple relationship to lower body mass (inset in Fig. 2B). The decreased number of nephrons and decreased capillary area with consequent hypofiltration could be associated with hyperfiltration in remnant healthy nephrons, where the increased intracapillary pressure would contribute to their late and progressive self-destruction [47], [48], [49] with late onset hypertension [50]. The onset of hypertension in older rats (aged 150 days) (Fig. 13) supports this view.

An increase in the glomerular area would be expected when the number of nephrons is decreased, and compensatory hyperfiltration occurs in some remnant glomeruli. It could be that intense collagen deposition in the cortex of programmed animals (Fig. 5) affects many glomerular structures (Fig. 5B), causing a global reduction in the size of the Bowman's capsule and glomerular capillary tufts (Fig. 4), with preservation or an increase in the area of others (likely to be hyperfiltrating glomeruli such as those marked in Fig. 5B). It is unexpected that GFR increases by 70% in 60 day-old programmed rats (Table 2), despite reduced glomerular areas as presented in Fig. 4. However, this could be explained by exacerbated hyperfiltration in the preserved nephrons. The intense proteinuria that accompanies the increase in GRF and U_Na_ (Table 2) reveals important and early damage in the filtration barrier that could evolve into global impairment of renal function in programmed rats. Therefore, kidneys from progeny that were programmed during lactation suffer from early severe morphological, and consequently functional, alterations in glomerular components, adding to the molecular alterations in proximal tubules, as discussed below.

Programmed rats had an augmented Na^+^ transport capacity in renal proximal tubule cells. Two alterations in the (Na^+^+K^+^)ATPase, which is considered responsible for the majority of Na^+^ reabsorption [17], [51], appeared to contribute to the doubling of its activity (Fig. 6A). Programming led to a significant increase in the expression of the predominant α_1_-catalytic subunit of this pump in renal tissue (Fig. 6B). Moreover, the molecular modification that caused a more than threefold increase in K^+^ affinity during the overall catalytic cycle (Fig. 7) could have contributed to accelerated turnover of the enzyme as higher K^+^ levels in the extracellular compartment increase the rate of the pacemaker step of the pumping cycle, the breakdown of the phosphorylated intermediate during the catalytic cycle [52]. Consequently, the increased pumping activity of proximal tubule (Na^+^+K^+^)ATPase will increase the transepithelial transport capacity. The extremely low K_0.5_ in the programmed group (0.5 mM) demonstrates that the pump becomes completely saturated and fully activated at physiological plasma K^+^ concentrations (5 mM). Undergoing accelerated cycles of Na^+^ transport through molecular alterations in the pump could compensate for the decreased activity of the local tissue renin/angiotensin system (RAS) in early life that is provoked by metabolic programming [53], [54], [55]. In addition to the well-known influence on distal segments, Ang II has an important stimulatory effect on transepithelial Na^+^ fluxes in proximal tubules [19] including that mediated by the (Na^+^+K^+^)ATPase [36]. Therefore, the constitutive up-regulation of (Na^+^+K^+^)ATPase could represent an adaptative response to a depressed tissue RAS, as discussed above.

It is plausible that an increase in single-nephron GFR contributes to up-regulated Na^+^-pumping activity in proximal tubule cells, as demonstrated for Na^+^∶K^+^∶2Cl^−^ transport in the thick ascending limb of pups exposed to prenatal low protein [56], which could lead to Na^+^ retention and the onset of hypertension in adult rats. However, the possibility of a compensatory natriuresis at 60 days of age in rats programmed during lactation is evident from the *in vivo* data presented in Table 2. In addition to the increase in GFR due to an over compensatory hyperfiltration in a population of glomeruli, the young adult programmed rats presented an increased U_Na_ (Table 2), possibly due to a pressure-natriuresis process, as recently proposed for low-protein prenatally programmed rats [57]. It is possible that Na^+^ retention, as a result of permanent up-regulation of Na^+^ pumps, is a late event in programmed rats when the onset of hypertension takes place. For this reason, the evolution of Na^+^ handling during a later stage is currently under study.

Maternal protein restriction during pregnancy is associated with fetal exposure to high glucocorticoid levels [58], and this can augment the Na^+^/H^+^ activity in luminal membranes [59], [60]. Therefore, an increased Na^+^ supply to (Na^+^+K^+^)ATPase could promote further stimulation of the pump and of transepithelial Na^+^ transport. However, in the case of protein restriction during lactation, maternal glucocorticoids are not modified with respect to normonourished rats [44], and the possible participation of the Na^+^/H^+^ exchanger in increased Na^+^ reabsorption remains an open question.

The furosemide-sensitive and ouabain-resistant Na^+^-ATPase activity is considered to be related to fine tuning of Na^+^ reabsorption in proximal tubules [17], [51]. This pumping activity has a different behavior and regulatory properties from (Na^+^+K^+^)ATPase [61], was partially characterized and purified [62], [63]. More recently [64], it was isolated, purified to homogeneity (molecular mass 89 kDa) and characterized, and it demonstrated the same biochemical behavior as that found in native membranes of kidney cells. In addition, the cDNA that codes for this pump was cloned, sequenced and silenced [64], giving support to the view that it is an independent enzymatic entity [61], [62], [63]. Its response to leptin and the proposed relationship of this ATP hydrolytic activity with dietary-induced and obesity-associated hypertension is of particular interest [17], [65]. It is also important that overactive renal ouabain-resistant Na^+^-ATPase resident in the basolateral membrane of proximal tubule cells, has been demonstrated in spontaneously hypertensive rats [23]. Programming has consequences for proximal tubule Na^+^-ATPase activity, which was also up-regulated (Fig. 8) and its physiological activation by Ang II – at a femtomolar concentration matching that found in plasma – was completely abolished (Fig. 9). The lack of effect of 10^−6^ M Ang II on Na^+^-ATPase activity in the control and the programmed groups deserves special consideration because the peptide inhibits transepithelial transport of fluid at this dose in microperfused proximal tubules [66]. This earlier observation could reflect the fact that in intact cells Ang II modulates signaling pathways [67] that influence (Na^+^+K^+^)ATPase in a biphasic manner [36].

The results depicted in Figs. 6, 7, 8, 9 demonstrate that programming during lactation strongly imprints the molecular machinery responsible for the majority of Na^+^ absorption and the fine tuning of this process, leading to augmented recovery of the filtered fluid, which can subsequently contribute to the onset of hypertension [15]. It is interesting that placental undernutrition promotes different modifications from those observed in the present study of young adult rats. In this context a diminution of (Na^+^+K^+^)ATPase activity with no change in the affinity for K^+^, and down-regulation of the ouabain-insensitive Na^+^-ATPase, are evident [18]. Recent observations [58] demonstrate that maternal protein restriction during gestation affects the expression of (Na^+^+K^+^)ATPase in a biphasic manner, with a decrease 12 days after birth and a significant increase in offspring aged 120 days. This supports the view that depending on the window of development, undernutrition may evoke different signals that affect the same organs in different ways [8].

Up-regulation of AT_1_ receptors and down-regulation of AT_2_ receptors could represent the persistence into adulthood of an adaptative response towards impaired RAS functioning in early life [53], [54], [55]. Lower local Ang II production in early life could be compensated by the programming-induced reciprocal alterations in the populations of AT_1_ and AT_2_ receptors as demonstrated in Fig. 10, which persisted into adult life. In contrast to the results concerning active Na^+^ transporters, both intrauterine and postnatal malnutrition promote the same patterns of Ang II receptor expression [18], [51]. It is interesting that maternal protein restriction during gestation leads to alterations in the expression of RAS components, with down-regulation of AT_1_ and AT_2_ receptors [58], [68], and possible loss of the inhibition of the (Na^+^+K^+^)ATPase by (high) Ang II [36]. These results give additional support to the idea that, depending on the period of development (and nephrogenesis; [8]), the impact of undernutrition on renal molecular targets will be varied, although late hypertension appears to be a final and common consequence [6], [12], [16], [53], [54], [55], [58], [68]. Treatment with losartan using gavage from weaning to 90 days of age prevents increases in Na^+^-ATPase activity and the onset of hypertension in a model of chronically undernourished rats (8% protein) (data not shown). Furthermore, losartan prevents a parallel increase in Na^+^-ATPase activity and arterial pressure found in spontaneously hypertensive rats [23]. These data provide support for the view that an early imbalance of Ang II receptors is involved in alterations to Na^+^-ATPase activity and the development of hypertension in adult offspring.

Programming affected the downstream PKC component of the signaling cascade that links AT_1_ receptors and the Na^+^-ATPase [20], [21]. Therefore, it is tempting to propose that the more than tenfold decrease in PKC activity (Fig. 11A) results in the lack of response of Na^+^-ATPase to Ang II (Fig. 9). PKC is located in the plasma membrane in a quiescent state [69], and the observation that there was only a moderate decrease in the levels (in contrast to activity) of the membrane-associated α and ε isoforms of the enzyme (Fig. 11B and 11C) supports the view that decreased turnover of primed PKC [69], rather than impaired targeting, is the result of programming during lactation. The comparable decreases in activity and expression of PKA in programmed rats (Fig. 12) indicates another possible cause for the increase in Na^+^-ATPase activity and possibly of the (Na^+^+K^+^)ATPase. We have previously demonstrated that Na^+^-ATPase is an effector for PKA in the basolateral membranes of kidney proximal tubule cells, coupled to AT_2_ in a way that antagonizes the effects mediated by AT_1_ receptors, leading to inhibition of the pump [37], and PKA participates in an intracellular signaling cascade that inactivates (Na^+^+K^+^)ATPase [36]. Therefore, global depression of the AT_2_ receptor→PKA pathway could be an adaptive response to decreased local activity of RAS promoted by undernutrition-induced metabolic programming [53], [54], [55].

The onset of late hypertension in the programmed group (Fig. 13) supports the view that the augmented Na^+^ transport (Figs. 6, 7, 8, 9) and the alterations of key components of its regulatory machinery (Figs. 10, 11, 12), together with the decreased number of nephrons and glomerular structural alterations (Figs. 3 and 4), are critical events caused through undernutrition during lactation that lead to adult hypertension and possible progression to renal disease. These events could also help in the elucidation of molecular mechanisms underlying the programming hypothesis (2, 3, 4, 5).

The observations presented in this work are novel in several respects as they elucidate − for the first time, to the best of our knowledge – the molecular targets of undernutrition during lactation that promote alterations in Na^+^ handling in the proximal tubule cells of adult offspring. The modifications described herein should aid in the understanding of how important ensembles of programmed events can contribute to the onset of hypertension in adult life [6], [50].
